# *Actinobacillus pleuropneumoniae* grows as aggregates in the lung of pigs: is it time to refine our *in vitro* biofilm assays?

**DOI:** 10.1111/1751-7915.12432

**Published:** 2016-10-28

**Authors:** Yannick D. N. Tremblay, Josée Labrie, Sonia Chénier, Mario Jacques

**Affiliations:** ^1^ Groupe de recherche sur les maladies infectieuses du porcFaculté de médecine vétérinaireUniversité de MontréalSt‐HyacintheQCJ2S 2M2Canada; ^2^ Laboratoire d’épidémiosurveillance animale du QuébecMinistère de l'Agriculturedes Pêcheries et de l'Alimentation du QuébecSt‐HyacintheQCJ2S 7X9Canada

## Abstract

*Actinobacillus pleuropneumoniae* causes porcine pleuropneumonia and forms biofilms *in vitro* on abiotic surfaces; however, presence of biofilms during infections has not been documented. The aim of this study was to use a species‐specific fluorescent oligonucleotide probe and confocal microscopy to localize *A. pleuropneumoniae* in the lungs of two naturally infected pigs. *Actinobacillus pleuropneumoniae* was detected by fluorescence *in situ* hybridization and observed to grow as aggregates (~30–45 μm) during a natural infection. As the *A. pleuropneumoniae* aggregates observed in porcine lungs differed from the biofilms grown on a solid surface obtained *in vitro*, we designed a new biofilm assay using agarose, a porous substrate, favouring the formation of aggregates. In this study, we described for the first time the mode of growth of *A. pleuropneumoniae* during a natural infection in pigs. We also propose an *in vitro* biofilm assay for *A. pleuropneumoniae* using a porous substrate which allows the formation of aggregates. This assay might be more representative of the *in vivo* situation, at least in terms of the size of the bacterial aggregates and the presence of a porous matrix, and could potentially be used to test the susceptibility of *A. pleuropneumoniae* aggregates to antibiotics and disinfectants.

## Introduction

Bacterial biofilms are structured clusters of bacterial cells enclosed in a self‐produced polymer matrix that are attached to a biotic or abiotic surface (Costerton *et al*., 1999; Jacques *et al*., 2010). This structure protects bacteria from hostile environmental conditions. Bacteria within a biofilm can resist attack from the host immune response, and are less sensitive than planktonic cells to the action of antibiotics and disinfectants.

*Actinobacillus pleuropneumoniae* is a Gram‐negative bacterium belonging to the *Pasteurellaceae* family and is the causative agent of porcine pleuropneumonia, a disease causing important economic losses to the swine industry worldwide (Gottschalk, 2012). Several virulence factors of *A. pleuropneumoniae* have been identified and these include the Apx toxins, iron uptake systems, adhesins and surface polysaccharides (Bossé *et al*., 2002; Chiers *et al*., 2010). We have shown that *A. pleuropneumoniae* is able to produce a dense biofilm on abiotic (plastic and glass; Labrie *et al*., 2010; Tremblay *et al*., 2013a) and biotic (cell line; Tremblay *et al*., 2013b) surfaces. We have also shown that *A. pleuropneumoniae* biofilm cells were 100–30 000 times more tolerant to ampicillin, florfenicol, tiamulin or tilmicosin than their planktonic counterparts (Archambault *et al*., 2012). Such decrease in susceptibility was dependent on the isolate. Additionally, we used whole‐genome DNA microarrays to identify differentially expressed genes in *A. pleuropneumoniae* planktonic cells or cells in biofilms formed under static (microtiter plate) or dynamic (drip‐flow biofilm reactor) conditions (Tremblay *et al*., 2013a).

Although the use of *in vitro* assays has greatly increased our understanding of the biology of bacterial biofilms, it is becoming increasingly apparent that many of these methods do not accurately represent *in vivo* conditions (Roberts *et al*., 2015). Thus, the purpose of this study was to use a species‐specific fluorescent oligonucleotide probe and confocal microscopy to localize *A. pleuropneumoniae* in the lungs of infected pigs.

## Results and Discussion

**Figure 1:**
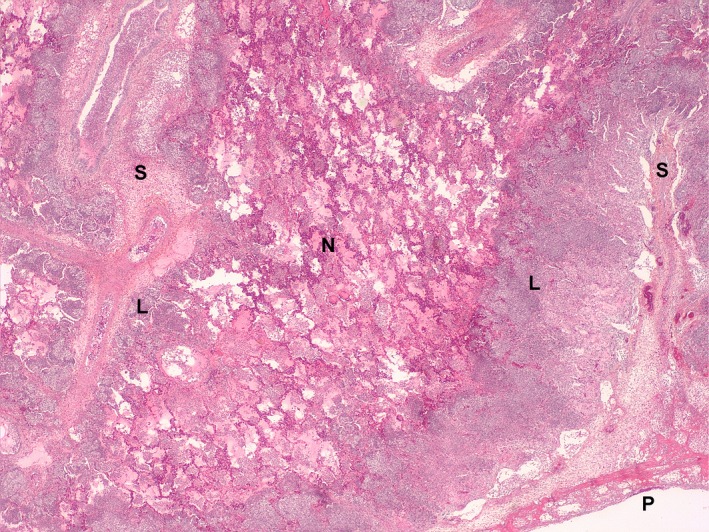
Swine lung, case SHY13‐04294. A large area of coagulation necrosis (N) that spares the interlobular septa, large vessels and airways (S) is present in the parenchyma, associated with oedema, congestion and fibrin exudation. It is surrounded by a rim of neutrophils and degenerated macrophages (L). Fibrin, oedema and leucocytes moderately distend the interlobular septa and pleura. The pleura (P) is covered by a thin layer of fibrin. Haematoxylin‐eosin‐phloxin‐saffron (HEPS), 25× magnification.

For this study, lungs were obtained from two pigs with clinical signs of infection and macroscopic examination consistent with acute porcine pleuropneumonia that were submitted to LEAQ (Laboratoire d’épidémiosurveillance animale du Québec). Microscopic examination of the lungs revealed typical, multiple foci of coagulation necrosis in the pulmonary parenchyma, associated with oedema, fibrin exudation, congestion, haemorrhages, capillary thrombosis, and the presence of microcolonies of small Gram‐negative bacilli. Numerous neutrophils and degenerated macrophages (round and oat cells) were delineating these foci. Fibrin, oedema, and leucocytes also severely distended the interlobular septa and pleura. The latter was covered with a thin layer of fibrin and leucocytes debris (Fig. 1). *Actinobacillus pleuropneumoniae* were isolated from the lung tissues of both pigs and were identified as APP4294 and APP286 for the isolates from the first and second pig respectively. Serotyping analysis (M. Gottschalk laboratory, Université de Montréal) showed that APP4294 and APP286 belong to serotype 7 and serotype 5, respectively, which are two of the most prevalent serotypes in North America (Gottschalk, 2012). Overall, the diagnostic tests confirmed that the animals were infected by *A. pleuropneumoniae* and that we were using lungs from natural infections.

**Figure 2:**
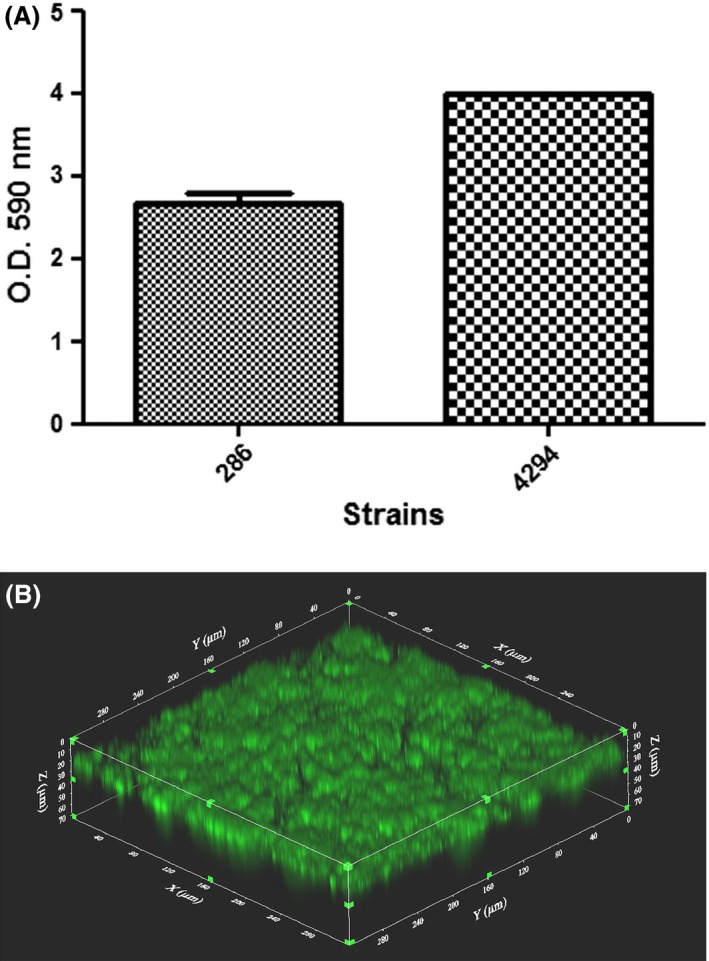
Biofilm formation by *Actinobacillus pleuropneumoniae* isolates APP286 and APP4294 in microtiter plates after 24 h of incubation. (A) OD_590 nm_ after crystal violet staining. (B) Confocal laser scanning microscopic image of biofilm of isolate APP286 stained with FilmTracer FM 1‐43. Stack of sections through the X‐Z plane is shown.

We first evaluated the capacity of these isolates to form biofilms *in vitro* in a standard microtiter plate assay routinely used in our laboratory. Both isolates formed a robust biofilm on the plastic surface after an incubation of 24 h (Fig. 2A). These biofilms were then visualized by confocal laser scanning microscopy. The biofilms completely covered the plastic surface and their thickness was evaluated to be around 50 μm (Fig. 2B).

**Figure 3:**
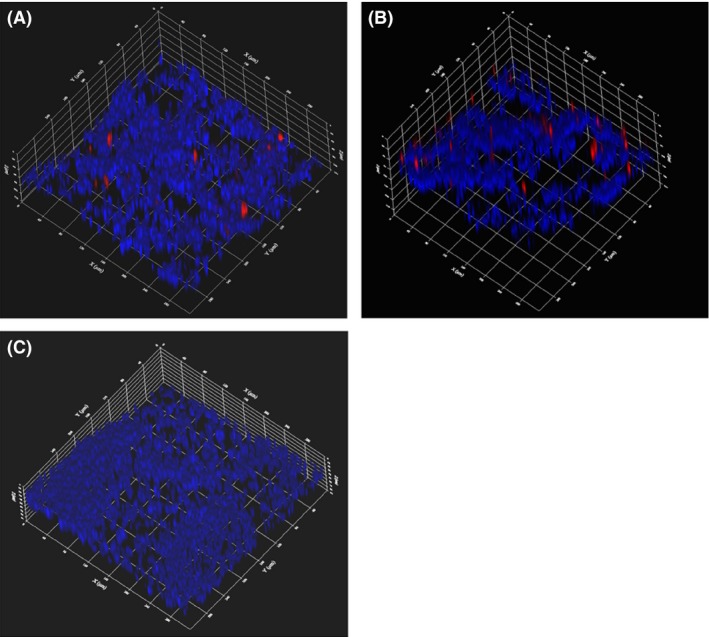
Fluorescence *in situ* hybridization (FISH) with an *apxIV* probe on the *Actinobacillus pleuropneumoniae*‐infected lung samples. CLSM images of *A. pleuropneumoniae* aggregates (red) within the lung tissue (blue) obtained from pigs of clinical cases SHY14‐286 (A) and SHY13‐04294 (B). (C) FISH on a lung sample from a control pig that was negative for *A. pleuropneumoniae*.

We then performed fluorescence *in situ* hybridization (FISH) with an *apxIV* probe on the lung samples. The use of species‐specific oligonucleotide probe allowed the localization of *A. pleuropneumoniae* cells (in red) within the lung tissue (stained with DAPI, in blue) (Fig. 3). It is clear from the images obtained from the lung of the pig infected with isolate of serotype 5 (Fig. 3A) that *A. pleuropneumoniae* seems to grow as aggregates (~30–45 μm) during a natural infection. An identical image was obtained from the lung of the other pig infected with a serotype 7 isolate (Fig. 3B). Controls (e.g. hybridization without probe, hybridization with probe of an *A. pleuropneumoniae* negative lung) (Fig. 3C) were performed to confirm the specificity of the FISH assay.

These aggregates are in agreement with recent reports in the literature indicating that the *in vivo* biofilms differ from their *in vitro* counterpart, in both size and shape (Roberts *et al*., 2015). Indeed, a meta‐analysis on the size of *in vivo* biofilms from human chronic infections by Bjarnsholt *et al*. (2013) showed the presence of bacterial aggregates ranging from ~5 to 200 μm in diameter; the median for the smallest and largest biofilm diameters were 5 and 50 μm respectively. Furthermore, these aggregates are not necessarily attached to a surface. Bjarnsholt *et al*. (2013) concluded that *in vivo* biofilms are smaller in physical dimensions, lack mushroom‐like structures, are embedded in host material and are continuously exposed to host defence reactions. More recently, Kragh *et al*. (2016) suggested that current models of biofilm formation should be reconsidered to incorporate the role of aggregates in biofilm initiation. In the case of *A. pleuropneumoniae* infections, lesion is known to contain blood, fibrin and leucocytes (dead or alive). Furthermore, neutrophils could release DNA during NET (neutrophil extracellular traps) formation and the extracellular DNA could help bacterial aggregation. We have previously observed that extracellular DNA increases biofilm formation and autoagregation in *A. pleuropneumaniae* (Hathroubi *et al*., 2015). Overall, these host components, combined to the lung architecture, could provide the porous substrate that supports the formation aggregates.

**Figure 4:**
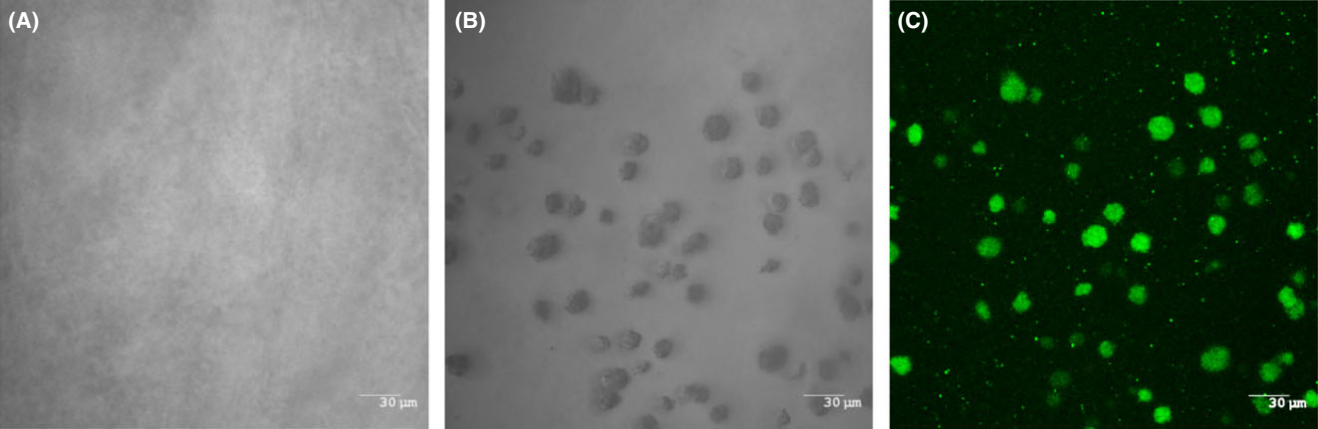
CLSM images of *Actinobacillus pleuropneumoniae* embedded in agarose after 24 h of incubation. (A) Agarose control, not inoculated with bacteria observed in the differential interference contrast mode. (B) *A. pleuropneumoniae* isolate APP286 aggregates as observed in the differential interference contrast mode, stack of ~10 images. (C) *A. pleuropneumoniae* isolate APP286 aggregates stained with WGA‐Oregon Green and observed in the fluorescence mode, stack of ~10 images. Bars = 30 μm.

As the *A. pleuropneumoniae* aggregates observed in porcine lungs differed from the biofilms grown on a solid surface obtained *in vitro*, we designed an *in vitro* assay using agarose, a porous substrate. The control, uninoculated agarose, showed a homogenous appearance when observed by differential interference contrast microscopy (Fig. 4A). *Actinobacillus pleuropneumoniae* grew as microcolonies or aggregates of approximately 20–30 μm when embedded in agarose (Fig. 4B), which is roughly similar in size to those observed in the porcine lungs. Furthermore, these aggregates were stained by the fluorescent lectin WGA‐Oregon Green 488 that binds to the poly‐*N*‐acetylglucosamine, a known component of the *A. pleuropneumoniae* biofilm matrix (Fig. 4C).

In this study, we described for the first time the mode of growth of *A. pleuropneumoniae* during a natural infection in pigs. We also propose an *in vitro* biofilm assay for *A. pleuropneumoniae* using a porous substrate which allows the formation of aggregates. This assay might be more representative of the *in vivo* situation, at least in terms of the size of the bacterial aggregates and the presence of a porous matrix, and could potentially be used to test the susceptibility of *A. pleuropneumoniae* aggregates to antibiotics and disinfectants.

## Experimental procedures

### Porcine lung samples and *Actinobacillus pleuropneumoniae* isolates

Two pigs with clinical signs and macroscopic and microscopic examinations consistent with acute porcine pleuropneumonia were submitted to LEAQ and used in the present study (clinical cases SHY13‐04294 and SHY14‐286). *Actinobacillus pleuropneumoniae* was isolated from the lung tissues of both pigs. The isolates were identified as APP4294 and APP286.

### *In vitro* biofilm assays

The two *A. pleuropneumoniae* isolates were grown on brain heart infusion agar or broth (BHI; Oxoid Ltd, Basingstoke, Hampshire, UK) supplemented with 5 μg ml^−1^ NAD (broth) or 15 μg ml^−1^ NAD (agar) (BHI‐NAD) at 37°C with 5% CO_2_. We evaluated their capacity to form biofilms *in vitro* in a standard microtiter plate assay routinely used in our laboratory (Labrie *et al*., 2010; Tremblay *et al*., 2013a). The biofilms were also visualized by confocal laser scanning microscopy (CLSM; FV1000 IX81; Olympus, Markham, ON, Canada) using FilmTracer FM 1‐43 (Molecular Probes, Eugene, OR, USA).

We also designed an *in vitro* assay using a porous substrate. *Actinobacillus pleuropneumoniae* isolates were grown overnight on BHI‐NAD plates as described above. A colony was transferred into 5 ml of BHI with 5 μg ml^−1^ NAD and incubated at 37°C overnight with agitation and then mixed (1:100) with agarose (autoclaved and placed in a water bath at 48°C) to obtain a final concentration of 0.5%. Aliquots of 1 ml were deposited in wells of a 24‐well microtiter plate; 1 ml of BHI‐NAD was added to each well once the agarose had solidified. The plates were then incubated at 37°C with 5% CO_2_ for 24 h. The agarose pellets were stained with Wheat Germ Agglutinin‐Oregon Green 488 (Molecular Probes) for 30 min at room temperature in the dark, then washed once and covered with PBS prior to observation by CLSM.

### Fluorescence *in situ* hybridization

We performed FISH with an *apxIV* probe on the lung samples as described by Loera‐Muro *et al*. (2013) with some modifications. Before the hybridization step, lung tissues were prepared and fixed as described by Niscito *et al*. (2009). Briefly, lungs were cut in small pieces (3 mm × 10 mm) and fixed with a solution of 4% paraformaldehyde‐PBS at 4°C for 6 h. Lungs were then washed two times for 10 min with PBS and kept at −20°C in 50% ethanol‐PBS until the hybridization. Lungs were put in Petri dishes and covered with 5–10 ml of hybridization solution. The hybridization solution contained 900 mM NaCl, 20 mM Tris‐Cl, 0.01% (v/v) SDS, 12.15% (v/v) formamide and 1.5 μM of Alexa Fluor 633‐labelled probe APXIVAN‐forward (GGGGACGTAACTCGGTGATT) (Invitrogen, Burlington, ON, Canada). Lung pieces were incubated for 18 h at 50°C and washed for 15 min at the same temperature with a solution containing 15 mM NaCl and 5 mM Trisma Base. The lung pieces were then rinsed in distilled water (50°C) and incubated for 5 min in a solution containing 0.1% (v/v) Triton X‐100. Lung pieces were washed three times with PBS and stained for 30 min at room temperature with DAPI. Two additional washes were done with PBS and the lungs pieces were transferred to a new Petri dish. PBS was added to fully submerged lung pieces and a glass slide was added on top to immobilize the lung pieces. The stained lung pieces were visualized by CLSM and images were acquired using the Fluoview software (Olympus).

## Conflict of interest

The authors declare no conflict of interests.
